# Susceptibility to e-cigarette among high school students: a study based on the ecological model of health behavior

**DOI:** 10.3389/fpubh.2024.1395717

**Published:** 2024-07-11

**Authors:** Hui Deng, Ling Fang, Lingyun Zhang, Jie Li, Jing Wang, Fan Wang, Pinpin Zheng

**Affiliations:** ^1^Department of Preventive Medicine and Health Education, School of Public Health, Institute of Health Communication, Key Lab of Public Health Safety of Ministry of Education, Fudan University, Shanghai, China; ^2^School of Journalism and Communication/National Media and Experimental Teaching Center, Jinan University, Guangzhou, Guangdong, China; ^3^General office, Pudong New Area Center for Patriotic Sanitation Campaign and Health Promotion Counsel, Shanghai, China; ^4^Fudan Development Institute, Fudan University, Shanghai, China

**Keywords:** susceptibility, e-cigarette, adolescent, tobacco control, risk factors

## Abstract

**Introduction:**

Youth e-cigarette (EC) use has rapidly increased in the last few years. It is crucial to identify the susceptible youth and prevent them from EC uptake. This study was conducted to investigate factors that affect youth susceptibility to EC use.

**Methods:**

This study employed a cross-sectional survey design, utilizing multi-center stratified cluster sampling method to select two junior high schools and two senior high schools in Shanghai, Guangzhou, and Chengdu. One-third of classes of each grade in the selected schools were involved in this survey. After obtaining the informed consent of parents, an anonymous and self-administered questionnaire was distributed to students. Questionnaire was designed based on the Ecological Models of Health Behavior. Associations between EC susceptibility and covariates were identified using multivariate logistic regression.

**Results:**

Among 2,270 students who had never vaped, 38.0% were susceptible to ECs. Logistic regression analysis identified factors on different levels affecting the susceptibility. Individual factors included senior high school students (OR = 1.34, 95% CI: 1.08–1.65), sensation seeker (OR = 1.11, 95%CI: 1.08–1.14), poor academic performance (OR = 1.24, 95% CI: 1.01–1.54), ever cigarette user (OR = 2.27, 95% CI: 1.29–4.01), unaware of the second-hand smoke from vaping (OR = 1.56, 95% CI: 1.25–1.96), agreeable with “I do not want to hang around vapers” (OR = 0.79, 95% CI: 0.64–0.97), agreeable with “ECs are more fashionable than cigarette” (OR = 2.50, 95% CI: 1.72–3.62) and favorable attitudes toward vaping (OR = 5.09, 95% CI: 3.78–6.85) were significantly associated with susceptibility to ECs. At interpersonal level, students who believe they would not be punished by parents for vaping increased susceptibility (OR = 1.27, 95% CI:1.01–1.59). At community level, exposure of EC advertising (OR = 1.81, 95% CI:1.46–2.25), exposure to hazard information (OR = 0.76, 95% CI: 0.59–0.97) and seeing vaping in daily life (OR = 2.11, 95% CI: 1.62–2.74), were statistically significantly associated with youth susceptibility to ECs.

**Conclusion:**

EC susceptibility was observed in a substantial proportion of adolescents who had never vaped, influenced by factors on different levels. This research underscores the urgent need for comprehensive intervention strategies to prevent the youth susceptibility to ECs.

## 1 Introduction

E-cigarette, also known as electronic nicotine delivery systems (ENDS), are devices designed to heat e-liquids to create aerosols which are inhaled by the user (1). These e-liquids usually contain glycerin, propylene glycol and flavorings, with or without nicotine. There’s a lot of controversies surrounding ECs. Some promoters believe that EC is a reduced harm alternative to cigarette (2) or a smoking cessation tool (3), while other opponents raise several concerns about ECs. Firstly, ECs can increase the risk of short-term adverse events including mouth and throat irritation, dry cough, and nausea (2), as well as the risk of long-term adverse events including asthma and COPD (4), brain damage (5), gastrointestinal, reproductive (6) and cardiovascular system damage (7, 8). Secondly, nicotine is a key addictive substance in e-liquids and has a bad impact on fetus, children and teenager’s brain and respiratory system development (9). Even those e-liquids who marked as containing “zero-nicotine” have been found contain nicotine when tested (10). Thirdly, a growing body of literature reports that ECs can be a gateway to cigarette initiation among nonsmokers (11).

The prevalence of EC use is higher among young people than in other age groups (12), of note, EC use among youth have shown a significant upward trend in the past few years. According to the Chinese National Youth Tobacco Survey, awareness of ECs among junior high school students rose from 45% in 2014 to 69.9% in 2019, while the prevalence of EC use increased from 1.2 to 2.7% over the same period (13, 14). The proportion of senior high school students who used ECs in the past 30 days was 2.2%, while the proportion for vocational school students was 4.5% (14). Further data from the 2021 Chinese National Youth Tobacco Survey found that 86.6% of students reported aware of EC. Additionally, the survey found that the prevalence of EC experimentation and current EC use among students was 16.1% and 3.6%, respectively (15). These figures underline a growing inclination toward EC experimentation and use among the youth, emphasizing the need to identify the susceptible youth and prevent them from EC uptake.

Susceptibility to EC usea strong independent predictor of experimentation (16–20), is defined as the absence of a firm decision not to use ECs (21). It is essential to identify adolescents who are susceptible to EC use to effectively direct prevention and intervention efforts. Factors identified by previous studies among adolescents include demographic factors [sex (16), age (16), and race/ethnicity (19)], psychological distress (22), sensation seeking traits (19), poor academic performance (19), tobacco (16) and alcohol (16) use, perception of little harm (19) and benefits (23) of EC, exposure to EC advertising (19), parent education (24), family members use tobacco (19), friends vape (25), perceiving vaping to be socially acceptable (25), believing ECs to be commonly used (23) and observing vaping at school (26).

There are two key limitations of prior work. Firstly, each of the studies focused only on a limited array of factors associated with susceptibility. This makes it difficult for intervention developers to determine the relative contribution of potential risk factors and where to focus their prevention efforts. Thus, a comprehensive exploration of potential risk factors is warranted. Secondly, the vast majority of research in this area has been conducted in the United States (18, 22, 26–28). Hence, there remains a gap in understanding the factors influencing EC susceptibility and use among Chinese youth. Some initial studies have begun to shed light on the issue, revealing that a considerable proportion of Chinese adolescents are at risk of becoming susceptible to EC use. A study, conducted in Tianjin in 2019, found that the proportion of susceptible teenagers was 15.8% (29). Despite this, comprehensive research on the susceptibility of Chinese youth to EC use remains scarce.

The Ecological Model of Health Behavior offers a multifaceted framework including individual, interpersonal, institutional, community and policy for understanding the complex interplay of factors at various levels that influence health behaviors (30). The first level, individual, focus on the knowledge, attitudes and skills related to the individual. The second level, interpersonal, deals with the exchanges and interactions within an individual’s network, encompassing both primary groups like family and close friends, and broader secondary groups. The organizational level targets social institutions that serve as established authorities and offer generally recognized and accepted purposes. The community level encompasses relationships that organizations form with each other. Finally, the public policy level contains all regulatory legislature, ranging from local municipalities to the central government. By integrating the Ecological Model of Health Behavior into the study of EC susceptibility among high school students, researchers can identify targeted strategies at each level of influence to effectively reduce the initiation and prevalence of EC use. Multilevel interventions, guided by the principles of the Ecological Model of Health Behavior, are likely to be more effective in changing behavior by addressing the complex and interconnected factors that contribute to EC susceptibility. This comprehensive approach not only aids in understanding the multifaceted nature of EC susceptibility among adolescents but also provides a solid foundation for the development of policies and practices aimed at its prevention and control.

## 2 Methods

### 2.1 Study design and participants

Between April and June 2021, a cross-sectional study employing anonymous pen-and-paper questionnaires was conducted among junior and senior high school students in Shanghai, Guangzhou, and Chengdu. These three cities are a well-known international metropolis and the economic center in the east, south and west of China. The rapid development in the area has increased awareness toward ECs among the local population.

A multi-center stratified cluster sampling method was utilized for this research. Schools in Shanghai, Guangzhou, and Chengdu were categorized by level (senior high school vs. junior high school) and further stratified based on the location and education quality. Following the principles of convenience sampling, two junior high schools and two senior high schools were selected in each city. Among each selected school, one-third of the classes of each grade were randomly chosen, and all students in these classes were provided with informed consent forms. After obtaining parental consent, students completed the self-administered and anonymous questionnaires in their classrooms. Study procedures were approved by the School of Public Health, Fudan University (IRB#2021-04-0898) as well as the participating schools.

### 2.2 Measures

#### 2.2.1 Dependent variables

The primary outcome of interest in this study was EC susceptibility, measured using methodologies aligned with those employed in prior research (18, 23, 31, 32). To assess susceptibility, adolescents were presented with three key questions: “Have you ever been curious about using ECs?,” “Do you think you will use ECs in the next 12 months?,” and “If one of your close friends offer you an EC, would you use it?” Response options for the first item included, “Not at all curious,” “A little curious,” “Somewhat curious,” or “Very curious”; response options for the remaining two items were, “Definitely not,” “Probably not,” “Probably yes,” or “Definitely yes.” Based on their responses, adolescents were classified as non-susceptible if they selected “Not at all curious” or “Definitely not” for all three questions. Conversely, they were considered susceptible if they chose any response other than above. Responses were marked as missing if any of the questions were not answered.

#### 2.2.2 Independent variables

The questionnaire was designed based on the Ecological Model of Health Behavior, incorporating three dimensions: individual factors, interpersonal factors, and community factors.

##### 2.2.2.1 Individual variables

***Sociodemographic characteristics:*** Demographics included city, school name, grade level (categorized as junior or senior), sex (categorized as male or female), mother education (categorized as below of senior high school, senior high school, or senior high school above).

***Sensation seeking:*** Sensation seeking has been linked to the initiation of cigarette and EC use (33–37). A sensation-seeking score was created from the Brief Sensation Seeking Scale (BSSS-4) (38): (1) “I would like to explore strange places,” (2) “I like to do frightening things,” (3) “I like new and exciting experiences, even if I have to break the rules,” and (4) “I prefer friends who are exciting and unpredictable.” Responses ranged from strongly agree (4) to strongly disagree (0), creating a mean score between 0 and 4.

***Academic performance:*** Participant were asked, “How was your overall grade ranked in your class last semester?” The responses were categorized into four distinct ranges: “1–25%,” “26–50%,” “51–75%,” and “Last 25%.”

***Tobacco use:*** Ever cigarette smoking was assessed with a single question: “Have you ever tried cigarette smoking, even one or two puffs?” (0 = No or 1 = Yes).

***Awareness of ECs:*** Awareness of ECs was gauged by asking if participants had heard of ECs prior to the survey (0 = No or 1 = Yes).

***Perception toward ECs:*** Five items were used to measure perceptions toward ECs, inquiring whether respondents believed that ECs are addictive, harmful to health, have second-hand smoke produced by vaping, have a safety risk, and have a negative impact on the cognitive development of teenagers (0 = unknown or 1 = known).

***Attitudes toward ECs:*** Three items were used to measure attitudes toward ECs. Respondents were asked to express their level of agreement with statements including “ECs are more fashionable than cigarettes” and “I do not want to hang around vapers,” with responses ranging from 1 (Strongly agree) to 5 (Strongly disagree). Additionally, we asked participants about their attitude toward vaping, with responses ranging from 1 (extremely like) to 5 (extremely dislike).

##### 2.2.2.2 Interpersonal variables

Influence from family were assessed by asking participants two questions. (1) “How many of your family members who lives with you now (mother, father, siblings, etc.) use ECs?.” Responses were coded as No (None) and Yes (At least one family member) due to its skewed distribution. (2) “Would your parents punish you if they caught you vaping?” “Definitely not,” with responses ranging from 1 (Definitely not) to 5 (Definitely yes).

Peer influence was assessed by asking, “How many of your five closest friends use ECs?,” with responses ranging from 0 (None) to 5 (5 out of 5 friends). We recoded this question to No (None) and Yes (At least one friend) due to its skewed distribution.

##### 2.2.2.3 Community variables

Participants were asked three questions: “How often have you seen EC advertisements in the past month?,” “How often have you seen information on the hazards of ECs in the past month?,” “How often have you seen others using ECs in daily life in the past month?.” Respondents could answer never, rarely, sometimes, often, or always. Participants were also asked whether they had seen ECs in stores around their school (0 = No, 1 = Yes).

### 2.3 Statistical analysis

Epidata 3.1 was used to input the data and SPSS v20.0 was used for all statistical analysis. Descriptive statistics about the distribution of variables are presented as frequency distributions and percentages, means and standard deviations. Multivariate logistic regression analysis was performed using three models to assess the influencing factors of adolescents’ EC susceptibility. Model 1 clarifies the impact of individual variables on adolescent EC susceptibility. Model 2 and Model 3 successively add interpersonal variables and community variables based on the previous model. Respondents with missing outcome values were excluded from both bivariate and multivariate analysis.

## 3 Results

### 3.1 Sample characteristics

This survey included a final sample of 12 public junior or senior high schools with a total sample size of 2,456 students. The analytic sample used in this study was students who had never used ECs (*n* = 2,270). Among students who had never used ECs, 34% were from Shanghai, 43% from Chengdu, and the remaining 24% from Guangzhou. Nearly 60% of the respondents were junior high school students, and 52% of the respondents were female. A small fraction (4%) tried cigarettes previously, and over 90% of the respondents were aware of ECs. Overall, 38% of students were susceptible to EC use.

Table 1 displays the distribution of characteristics across individual, interpersonal, and community levels between those susceptible and not susceptible to ECs. Students were significantly more susceptible to ECs if they were ever use cigarette (*p* < 0.001), aware of ECs (*p* < 0.001), poor academic performance (*p* < 0.001), no pocket money (*p* = 0.021), higher level of sensation seeking (*p* < 0.001), unaware that vaping can produce second-hand smoke (*p* < 0.001), unaware the addiction of ECs (*p* < 0.001), unaware the harm of ECs (*p* < 0.001), unaware the accident risk of ECs (*p* < 0.001), unaware of the negative impact of ECs on teenager’s cognitive development (*p* < 0.001), perceiving that ECs are more fashionable than cigarettes (*p* < 0.001), disagreeable with “I do not want to hang around vapers” (*p* < 0.001), and had best friends who vape (*p* < 0.001), often or always seeing vaping in daily life (*p* < 0.001), often or always seeing ECs advertising (*p* < 0.001) and never or seldom seeing ECs hazard information (*p* = 0.001).

**Table 1:** Individual, interpersonal, and community factors characteristics of high school students who had never used ECs.

Variable	Total	E-cigarette susceptibility		*p-*value
	*N* (%)/Mean ± SD	Yes	No	
**Individual factors**				
City				0.363
Shanghai	761 (33.52)	298 (39.16)	463 (60.84)	
Chengdu	966 (42.56)	351 (36.34)	615 (63.66)	
Guangzhou	543 (23.92)	214 (39.41)	329 (60.59)	
Grade level				0.272
Junior	1,293 (56.96)	479 (37.05)	814 (62.95)	
Senior	977 (43.04)	384 (39.30)	593 (60.70)	
Gender				0.133
Male	1,088 (47.93)	431 (39.61)	657 (60.39)	
Female	1,182 (52.07)	432 (36.55)	750 (63.45)	
Ever tried cigarettes				<0.001
No	2,185 (96.38)	807 (36.93)	1,378 (63.07)	
Yes	82 (3.62)	55 (67.07)	27 (32.93)	
Awareness of EC				<0.001
No	134 (5.91)	21 (15.67)	113 (84.33)	
Yes	2,134 (94.09)	841 (39.41)	1,293 (60.59)	
Maternal education				0.247
Below of senior high school	416 (18.65)	172 (41.35)	244 (58.65)	
Senior high school or equivalent	788 (34.71)	287(36.42)	501(63.58)	
Senior high school above	1,027 (45.24)	391 (38.07)	636(61.93)	
Self-report academic performance ranking				<0.001
Top 50%	1,495 (66.03)	529 (35.28)	966 (64.62)	
Last 50%	769 (33.97)	332 (43.17)	437 (56.83)	
Pocket money				0.021
Yes	1,678 (74.84)	660 (39.33)	1,018 (60.67)	
No	564 (25.16)	191 (33.87)	373 (66.13)	
Sensation seeking	2.43 ± 0.94	2.71 ± 0.91	2.26 ± 0.92	<0.001
Aware the addiction of ECs				<0.001
Yes	1983 (87.59)	707 (35.65)	1,276 (64.35)	
No	281 (12.41)	152 (54.09)	129 (45.91)	
Aware the harm of ECs				<0.001
Yes	2053 (90.64)	744 (36.24)	1,309 (63.76)	
No	212 (9.36)	116 (54.72)	96 (45.28)	
Aware the second-hand smoke produced by vaping				<0.001
Yes	1,589 (70.25)	528 (33.23)	1,061 (66.77)	
No	673 (29.75)	332 (49.33)	341 (50.67)	
Aware the accident risks of ECs				<0.001
Yes	1982 (87.47)	699 (35.27)	1,283 (64.73)	
No	284 (12.53)	162 (57.04)	122 (42.96)	
Aware the negative impact of ECs on the cognitive development				<0.001
Yes	1841 (81.24)	635 (34.49)	1,206 (65.51)	
No	425 (18.72)	226 (53.18)	199 (46.82)	
Believe that ECs are more fashionable than cigarettes				<0.001
Equally or less	2073 (91.69)	728 (35.12)	1,345 (64.88)	
More fashionable	188 (8.31)	128 (68.09)	60 (31.91)	
Agree with “I do not want to hang around vapers”				<0.001
Disagree/strongly disagree	931 (41.21)	439 (47.15)	492 (52.85)	
Agree/strongly agree	1,328 (58.79)	418 (31.48)	910 (68.52)	
Attitudes toward vaping				<0.001
Dislike or strongly dislike	1888 (83.25)	565 (29.93)	1,323 (70.07)	
Like or strongly like	380 (16.75)	297 (78.16)	83 (21.84)	
**Interpersonal factors**				
Family members vaping				0.204
No	2,155 (95.06)	814 (37.77)	1,341 (62.23)	
Yes	112 (4.94)	49 (43.75)	63 (56.25)	
Five best friends vaping				<0.001
No	2,171 (95.81)	802 (36.94)	1,369 (63.06)	
Yes	95 (4.19)	60 (63.16)	35 (36.84)	
Perceiving parental attitudes toward children vaping				<0.001
Punitive	1,634 (72.01)	558 (34.15)	1,076 (65.85)	
Non-punitive	635 (27.99)	304 (47.87)	331 (52.13)	
**Community factors**				
Frequency of seeing vaping in the daily life				<0.001
Not or rarely	538 (23.75)	116 (21.56)	422 (78.44)	
Often or always	1727 (76.25)	744 (43.08)	983 (56.92)	
Frequency of EC advertising exposure				<0.001
Not or rarely	1,536 (67.67)	486 (31.64)	1,050 (68.36)	
Often or always	734 (32.33)	377 (51.36)	357 (48.64)	
Frequency of EC hazard message exposure				0.001
Not or rarely	1744 (77.61)	696 (39.91)	1,048 (60.09)	
Often or always	503 (22.39)	161 (32.01)	342 (67.99)	
EC exposure in store nearby school				<0.001
Yes	318 (14.10)	161 (50.63)	157 (49.37)	
No	1938 (85.90)	697 (35.96)	1,241 (64.04)	

SD, standard deviation.

### 3.2 Multivariate logistic regression analyses of influencing factors of adolescents’ EC susceptibility

Table 2 shows the results of multivariate logistic regression analyses of influencing factors of students’ EC susceptibility (susceptible vs. insusceptible). In Model 1, we analyzed the associations between individual variables and EC susceptibility. Senior high school students showed a higher susceptibility compared to their junior high counterparts (OR = 1.28, 95% CI: 1.04–1.57). Ever cigarette use increased the susceptibility to ECs (OR = 2.76, 95% CI: 1.58–4.81). Students who self-reported academic score ranks last 50% in the class were more likely to be susceptible (OR = 1.25, 95% CI: 1.02–1.54). Teenagers with daily pocket money had a higher susceptibility to ECs (OR = 1.28, 95% CI: 1.01–1.62). High sensation-seeking adolescents were more prone to EC use (OR = 1.12, 95%CI: 1.09–1.15). Adolescents with a favorable attitude toward vaping (OR = 6.35, 95% CI: 4.74–8.52) were more susceptible to ECs. Those who are unaware of the second-hand smoke produced by vaping increased susceptibility to ECs (OR = 1.51, 95% CI: 1.19–1.91). Teenagers who believe ECs are more fashionable than cigarettes were more susceptible (OR = 2.88, 95% CI: 1.97–4.20). Adolescents who agree with “I do not want to hang around vapers” were less susceptible to ECs (OR = 0.78, 95% CI: 0.63–0.96). Model 2 showed that adolescents who do not believe their parents would not punish them for using ECs were more susceptible (OR = 1.22, 95% CI: 1.01–1.50). Model 3 revealed the associated factors on community level. Adolescents who frequently observe people vaping in daily lives were more susceptible to ECs (OR = 2.11, 95% CI: 1.62–2.74). Frequently exposure to EC advertisements increased susceptibility (OR = 1.81, 95% CI: 1.46–2.25). Adolescents who frequently see information about the harms of ECs were less susceptible (OR = 0.76, 95% CI: 0.59–0.97).

**Table 2:** Multivariate logistic regression on influencing factors of e-cigarette susceptibility among high school students who never used ECs.

Variable	Model 1	Model 2	Model 3
	OR (95%CI)	OR (95%CI)	OR (95%CI)
**Individual factors**			
**City**			
Shanghai	Referent		
Chengdu	1.14 (0.87–1.49)	1.14 (0.87–1.48)	1.11 (0.85–1.46)
Guangzhou	1.00 (0.77–1.29)	1.02 (0.78–1.16)	0.95 (0.73–1.23)
**School type**			
Junior high school	Referent		
Senior high school	1.28 (1.04–1.57)^∗^	1.28 (1.05–1.58)^∗^	1.34 (1.08–1.65)^∗∗^
**Gender**			
Male	Referent		
Female	0.91 (0.74–1.12)	0.95 (0.78–1.16)	1.07 (0.87–1.32)
**Ever tried cigarettes**			
No	Referent		
Yes	2.76 (1.58–4.81)^∗∗∗^	2.76 (1.58–4.81)^∗∗^	2.27 (1.29–4.01)^∗∗^
**Self-report academic score ranking**			
Top 50%	Referent		
Last 50%	1.25 (1.02–1.54)^∗^	1.26 (1.03–1.55)^∗^	1.24 (1.01–1.54)^∗^
**Number of pocket money**			
0	Referent		
>1	1.28 (1.01–1.62)^∗^	1.28 (1.01–1.61)^∗^	1.14 (0.90–1.45)
Sensation seeking	1.12 (1.09–1.15)^∗∗∗^	1.11 (1.08–1.14)^∗∗∗^	1.11 (1.08–1.14)^∗∗∗^
**Attitudes toward vaping**			
Dislike or strongly dislike	Referent		
Like or strongly like	6.35 (4.74–8.52)^∗∗∗^	6.13 (4.59–8.18)^∗∗∗^	5.09 (3.78–6.85)^∗∗∗^
**Aware of the second-hand smoke produced by vaping**			
Yes	Referent		
No	1.51 (1.19–1.91)^∗∗^	1.57 (1.26–1.94)^∗∗∗^	1.56 (1.25–1.96)^∗∗∗^
**Believe that ECs are more fashionable than cigarettes**			
Equally or less	Referent		
More fashionable	2.88 (1.97–4.20)^∗∗∗^	2.81 (1.95–4.04)^∗∗∗^	2.50 (1.72–3.62)
**Agree with “I do not want to hang around vapers”**			
Disagree/strongly disagree	Referent		
Agree/strongly agree	0.78 (0.63–0.96)^∗^	0.78 (0.64–0.96)^∗^	0.79 (0.64–0.97)^∗^
**Interpersonal level**			
**Family members vaping**			
No		Referent	
Yes		1.36 (0.86–2.17)	1.56 (0.98–2.48)
**Closest friends vaping**			
No		Referent	
Yes		0.69 (0.41–1.15)	0.82 (0.49–1.39)
**Perceiving parental attitudes toward children vaping**			
Punitive		Referent	
Non-punitive		1.22 (1.01–1.50)^∗^	1.27 (1.01–1.59)^∗^
**Community level**			
**Frequency of seeing vaping in the daily life**			
Low			Referent
High			2.11 (1.62–2.74)^∗∗∗^
**Frequency of e-cigarette advertising exposure**			
Not or rarely			Referent
Often or always			1.81 (1.46–2.25)^∗∗∗^
**Frequency of e-cigarette hazards message exposure**			
Not or rarely			Referent
Often or always			0.76 (0.59–0.97)^∗^

OR, odd ratios; 95% CI, 95% confidence intervals. ^∗^*p* ≤ 0.05; ^∗∗^*p* ≤ 0.01; ^∗∗∗^*p* ≤ 0.001.

## 4 Discussion

To our knowledge, this study is one of the first studies to explore a comprehensive set of multilevel determinants affecting adolescent susceptibility to ECs, based on the Ecological Model of Health Behaviors. A substantial proportion (38%) of the adolescents who had never smoked were found to be susceptible to future vaping. The results of logistic regression analysis showed that demographics (senior high school students, sensation seeking, poor academic performance, ever cigarette user), poor knowledge about ECs (unaware of second-hand smoke produced by vaping), and approbatory attitudes toward EC (disagreeable with “I do not want to hang around vapers,” agreeable with “ECs are more fashionable than cigarette,” favorable attitudes toward vaping) increased susceptibility. Also, interpersonal factors (parental norms regarding children EC use) and community factors (exposure of EC advertising and hazard information, seeing vaping in daily life) were statistically significantly associated with youth susceptibility to EC initiation. Given susceptibility is a strong predictor of subsequent use, efforts are urgently needed to reduce susceptibility among young Chinese to protect them from future uptake.

### 4.1 Individual factors

Aligned with prior research, our findings confirmed that individual predictors of EC susceptibility are similar to the earlier studies. Our research showed that senior high school students were more susceptible to ECs than junior high school students. As such, our result echoes the findings of the China National Youth Tobacco Survey. This survey reported a higher prevalence of EC use among senior high students (15). Contrary to the findings of several previous studies (16, 18, 19, 22, 25–27, 39, 40), our study did not observe a significant gender difference in susceptibility to ECs, indicating the necessity for intervention strategies that are inclusive of all genders. Additionally, different from the previous studies (24, 41, 42), our study found no significant differences in EC susceptibility based on parental education levels. Consistent with earlier studies (19, 22), students with poor academic performance were more susceptible to ECs. In line with previous investigations (19, 23, 25, 43), our study also found that sensation seeking was a significant factor associated with increased susceptibility to ECs among adolescents. Given that sensation seeking has been established as a strong predictor of various substance use and risky behaviors in adolescents (44), developing targeted interventions to address modifiable risk factors among individuals prone to high sensation seeking. Such interventions may serve to reduce the number of susceptible youths to ECs, and potentially decrease the number of EC user.

Individual knowledge, attitudes and behaviors were also associated with susceptibility to ECs. Our survey revealed that a considerable awareness among participants regarding the health risks of ECs. Over 90% of participants acknowledged the health hazards, 88% recognized the addiction, 87% were aware of associated accident risks, and 81% understood their negative impact on adolescent cognitive development. However, only about 70% were informed about the second-hand smoke produced by vaping. Hence, as youth knowledge evolves, targeted awareness campaigns can be effectively developed, such as focusing on the risks of second-hand smoke from vaping. The belief that ECs are more fashionable than cigarettes and favorable attitudes toward vaping were associated with increased susceptibility, whereas a reluctance to hang around vapers served as a protective factor. These beliefs may be influenced by EC companies’ marketing strategies. A content analysis revealed that Chinese EC manufacturers often promote their products with appealing words, highlighting supposed health benefits (e.g., reduced harm), emotional advantages (e.g., positive mood), and lifestyle benefits (e.g., friendship/love) (45). Therefore, interventions using tailored messages to counteract misleading and exaggerated marketing about ECs may be essential in preventing susceptibility. As expected, ever cigarette use increased the EC susceptibility among students, consistent with prior studies (16, 26, 28, 39, 40). Given that susceptibility to ECs serves as a strong independent predictor of experimentation, this underscore the necessity for integrated and comprehensive prevention strategies.

### 4.2 Interpersonal factors

Previous studies (19, 24, 41) have established a correlation between the use of tobacco (include cigarettes and ECs) by family members and increased susceptibility to EC use among adolescents. About 5% of the respondents reported vaping within their family. Although our survey did not find an association between family members’ EC use and student susceptibility, it’s evident that the family environment plays a significant role in adolescents’ susceptibility to EC use. Factors such as smoke-free household environment (41), not imposing curfew (41), and restrictions on media use (43) were associated with EC susceptibility among teenagers. Our survey further identified a relationship between parenting norms regarding EC use and adolescents’ susceptibility to them. Students who perceived a lack of punitive consequences from their parents for EC use were found to be more susceptible. This underlines the importance for parents to be aware of the powerful impact their attitudes and behaviors can have on their children’s inclination toward ECs. Parents are encouraged to adopt a strict no-use policy, refrain from using cigarettes and ECs themselves, and foster a smoke-free environment at home.

Nearly 96% of the respondents reported that none of their five best friends vaped. Our research did not find a peer use different in susceptibility, which diverges from several studies (25, 27, 40). This could reflect a unique aspect of the surveyed population or indicate a shift in the social dynamics surrounding vaping.

### 4.3 Community factors

Consistent with multiple literatures (19, 24–27, 46), we found that exposure to EC advertising was correlated with an increased risk of EC use among youth. This highlights the potential benefits of stricter regulations on EC advertising in China, which could reduce adolescents’ exposure to marketing and decrease susceptibility among non-users. Our study revealed an association between adolescents’ exposure to information on the hazards of ECs and their susceptibility to using ECs. Notably, while 32% of adolescents were frequently exposed to EC advertisements, only 22% were regularly exposed about the health risks associated with ECs. This disparity highlights a significant gap in the dissemination of essential health information regarding ECs, potentially leading to an underestimation of their associated risks among the public. This observation also highlights the urgent need for enhanced efforts in public education and awareness, particularly focused on spreading comprehensive information about the harms of ECs. It’s imperative for governments and public health institutions to take action toward increasing the visibility of EC health risks. Strategies could include amplifying the availability of educational resources which details the health risks associated with EC use. By implementing these measures, there is an opportunity to significantly improve public understanding of EC hazards, empowering individuals, especially the youth, to make more informed health decisions.

Our research identified a clear association between the frequent observation of vaping in daily life and increased susceptibility to EC use among youth. This finding is consistent with other studies indicating that adolescents who witness EC use at school (26), believe ECs to be commonly used (23) and perceive the social acceptability of ECs (25) are more susceptible to EC use. Seeing vaping in daily life would normalize EC use, promote social acceptance of ECs, and undermine efforts to maintain a smoke-free environment. As of March 2024, 24 cities have enacted local smoke-free legislation, with 9 of these cities including ECs within their smoke-free laws (47). Therefore, there is a need to advocate for the inclusion of ECs in smoke-free legislation in the future.

The findings of this study illuminate potential paths for future interventions aimed at mitigating the susceptibility to ECs among adolescents. Given the high level of awareness about ECs among both junior and senior school students, it is imperative for educational institutions to incorporate EC education into existing smoking prevention programs. There is a critical need for health education and promotion activities that clearly communicate the risks and harms associated with EC use. Monitoring adolescent perceptions, attitudes, and behaviors over time is essential, allowing for the development of targeted interventions based on these observations. Special focus should be placed on groups identified as being at higher risk, including senior high school students, those identified as sensation seekers, students with poor academic performance, those who have previously used cigarettes, and students who perceive fewer harms and more benefits from EC use. Parents play a crucial role in influencing their children’s attitudes toward ECs. It is vital for parents to set clear rules against the use of ECs and lead by example by not using cigarettes or ECs themselves. Moreover, government agencies are tasked with enforcing existing laws more rigorously, enhancing regulations surrounding ECs, and closely monitoring EC advertising and marketing practices. To further protect the youth, it is essential to create a smoke-free environment for the adolescents. Banning the sale of ECs to minors, prohibiting their advertisement, and restricting their sale in stores near schools are critical steps.

### 4.4 Limitation and strength

The notable strengths of this study are derived from its foundation in the Ecological Model of Health Behaviors, offering a comprehensive exploration of the factors influencing susceptibility to ECs among junior and senior high school students in three developed cities. Additionally, the use of a validated measure for assessing EC susceptibility (18, 23) increase credibility to our findings, providing scientifically grounded suggestions for the development of future prevention and control policies and contributing valuable insights for subsequent research in this area.

However, our study is not without its limitations. Firstly, being a cross-sectional study, it cannot determine the causality or temporal sequence of the observed associations. The self-reported questionnaires also introduce the possibility of recall and reporting biases. Additionally, there is the potential for social desirability bias, where participants might provide responses that they believe are more socially acceptable rather than their true behaviors or opinions. This could particularly affect sensitive topics such as e-cigarette use, potentially leading to underreporting or overreporting. Secondly, the influence of policy factors on adolescents’ susceptibility to ECs was not examined. This was largely due to the uniformity in EC regulatory policies across the three surveyed cities at the time of our investigation. Thirdly, this study was only conducted in three provincial capitals/municipalities, and did not investigate the situation in small and medium-sized cities and rural areas, so the generalizability of the conclusions may be somewhat limited. Different socio-cultural contexts in these areas could lead to different patterns of behavior and attitudes toward ECs. Therefore, our findings may not be fully applicable to other populations or regions with different socio-cultural contexts. Future studies should include a more diverse range of locations to enhance the external validity of the findings and provide a more comprehensive understanding of the issue.

## 5 Conclusion

A substantial proportion of adolescents who had never vaped showed a susceptibility to EC. Based on the Ecological Model of Health Behavior, our study identified the associated factors of EC susceptibility. Individual variable included senior high school students, sensation seeker, poor academic performance, ever cigarette user, a lack of knowledge about EC harm (unaware of second-hand smoke produced by vaping), and positive attitudes toward EC (disagreeable with “I do not want to hang around vapers,” agreeable with “ECs are more fashionable than cigarette,” favorable attitudes toward vaping) increased susceptibility. Furthermore, interpersonal factors (parental norms regarding children EC use) and community factors (exposure of EC advertising and hazard message, seeing vaping in daily life) were statistically significantly associated with youth susceptibility to ECs. Overall, our findings highlight the critical need for intervention strategies that address individual, interpersonal, and community-level factors.

## Data availability statement

The datasets generated for this study are available on request to the corresponding author.

## Ethics statement

The studies involving humans were approved by the School of Public Health, Fudan University. The studies were conducted in accordance with the local legislation and institutional requirements. Written informed consent for participation in this study was provided by the participants’ legal guardians/next of kin.

## Author contributions

HD: Formal analysis, Writing – original draft, Methodology, Investigation, Conceptualization. LF: Writing – review & editing, Data curation. LZ: Writing – review & editing, Data curation. JL: Data curation, Writing – review & editing, Investigation. JW: Writing – review & editing, Data curation, Investigation. FW: Data curation, Writing – review & editing, Investigation, Conceptualization. PZ: Writing – review & editing, Project administration, Methodology, Investigation, Funding acquisition, Formal analysis, Data curation, Conceptualization.
